# Extending the Calgary Audit and Feedback Framework into the virtual environment: a process evaluation and empiric evidence

**DOI:** 10.1186/s43058-024-00679-5

**Published:** 2024-12-18

**Authors:** Douglas Woodhouse, Diane Duncan, Leah Ferrie, Onyebuchi Omodon, Ashi Mehta, Surakshya Pokharel, Anshula Ambasta

**Affiliations:** 1 Physician Learning Program, University of Calgary, Calgary, Canada; 2 Department of Family Medicine, Cumming School of Medicine, University of Calgary, Calgary, Canada; 3 Department of Medicine, Cumming School of Medicine, University of Calgary, Calgary, Canada; 4 Health Quality Council of Alberta, Calgary, Canada; 5 Ward of the 21st Century, University of Calgary, Calgary, Canada; 6 Department of Anesthesia, Pharmacology and Therapeutics, University of British Columbia, Vancouver, Canada

**Keywords:** Audit and feedback, Virtual facilitation, Quality Improvement, Health care waste, Low value testing, Social learning

## Abstract

**Background:**

The Calgary Audit and Feedback Framework (CAFF) is a pragmatic, evidence-based approach for the design and implementation of *in-person* social learning interventions using Audit and Group Feedback (AGF). This report describes extension of CAFF into the virtual environment as part of a multifaceted intervention bundle to reduce redundant daily laboratory testing in hospitals. We evaluate the process of extending CAFF in the virtual environment and share resulting evidence of participant engagement with planning for practice change.

**Methods:**

We describe an innovative virtually facilitated AGF intervention based on the CAFF. The AGF intervention was part of an intervention bundle which included individual physician laboratory test utilization reports and educational tools to reduce redundant daily laboratory testing in hospitals. We used data from recorded and transcribed virtual AGF sessions, post AGF session surveys and detailed field notes maintained by project team members. We used simple descriptive statistics for quantitative data and analyzed qualitative data according to the elements of CAFF.

**Results:**

Eighty-three physicians participated over twelve virtual AGF sessions conducted across four tertiary care hospitals during the study period. We demonstrate that all prerequisite activities for CAFF (relationship building, question choice and data representation) were present in every virtual AGF session. Virtual facilitation was effective in supporting the transition of participants through different steps of CAFF in each session to lead to change talk and planning. All participants contributed to discussion during the AGF sessions. The post AGF session surveys were filled by 66% of participants (55/83), with over 90% of respondents reporting that the session helped them improve practice. 46% of participants (38/83) completed personal commitment to change forms at the end of the sessions.

**Conclusions:**

Virtual AGF sessions, developed and implemented with fidelity to the CAFF approach, successfully engaged physicians in a group learning environment that led to change planning. Further studies are needed to determine the generalizability of our findings and to add to the literature on evidence-based virtual facilitation techniques.

**Supplementary Information:**

The online version contains supplementary material available at 10.1186/s43058-024-00679-5.

Contributions to the literature

- There is heterogeneity in the methodologies used to conduct Audit and Feedback studies for healthcare practice improvement in the literature.
- Our study extends an existing evidence-based approach for design of Audit and Group Feedback called Calgary Audit and Feedback Framework (CAFF) into the virtual environment with a detailed description of the virtual facilitation techniques used.
- Through a comprehensive process evaluation, we demonstrate that the elements of CAFF can be reliably reproduced in the virtual environment.
- Our results build upon the empirical evidence base for CAFF and for virtual facilitation in general.

## Introduction

Active de-implementation efforts are needed to reduce low-value care practices such as repetitive laboratory testing in hospitalized patients [1–5]. Multi-faceted interventions with education and feedback components are effective in reducing laboratory test over-utilization [6, 7]. However, there is heterogeneity in the methodologies used to deliver audit and feedback. While literature indicates that feedback is more effective when it is provided more than once, in both verbal and written formats, by a supervisor or colleague, and with explicit targets and an action plan; only few studies feature these best practices [8, 9]. In an effort to advance the science of audit and feedback in healthcare, experts advocate for the use of theory-based design of audit and feedback that draws from the domains of implementation science, motivation and behaviour change theory, and the educational feedback literature [10]. Building on these domains, Brehaut et al*.* have published recommendations to enhance the effectiveness of feedback which include attention to topic choice, provision of individualized data with relevant comparators, integration of summary messages and data visualization, management of cognitive load and use of social interaction to construct feedback [11].

The Calgary Audit and Feedback Framework (CAFF) is a practical evidence-informed approach for the design and implementation of facilitated audit and group feedback (AGF) based on implementation science, motivational and behavior change theory, and educational feedback literature as described by Colquhoun et al. [10] The theoretical design of CAFF AGF sessions borrows from iPARIHS framework [12], recommendations from Brehaut et al. [11], social learning theory [13], and the R2C2 model [14]. In-person CAFF AGF sessions include one physician facilitator, one peer leader and up to 20 participants in one room, with an agenda that builds in relationship building, and emphasizes importance of question choice. Participants review reports containing their own individualized performance data and are co-facilitated to identify opportunities, barriers and enablers for change [15]. Participants move through a cycle of discrete behaviours (reacting to data, understanding and questioning, justifying and contextualizing, and reflecting, sharing practices and discussing evidence for best practices) towards the end goal of planning for change [15]. CAFF has been efficaciously applied to support AGF sessions amongst different participant groups, including emergency medicine physicians [16], rheumatologists [17], cardiologists [18], anesthesiologists [19] and critical care physicians [20].

The COVID-19 pandemic necessitated extension of CAFF-based AGF sessions into the virtual setting. Virtual facilitation is becoming increasingly common as an implementation strategy, but there exists variability on how virtual facilitation is reported [21]. It was unclear if virtually facilitated CAFF would be effective in building the relationships and trust required to engage groups of physicians in social interaction and behaviours leading to change talk and planning [15, 21]. Implementing this intervention without fidelity may undermine its effectiveness [22, 23]. In this manuscript, we describe the development and delivery of a novel virtual AGF session, demonstrate fidelity to CAFF elements through a process evaluation, and establish that virtual facilitation allowed participants to plan for practice change.

## Methods

We followed the SQUIRE reporting guidelines [24] to provide rigour to this quality improvement report (Appendix 1). The Calgary Physician Learning Program [25] (that focuses on development and delivery of AGF sessions) partnered with a quality improvement initiative called Re-Purposing the Ordering of Routine laboratory Tests (RePORT) to reduce the overutilization of laboratory testing in hospitalized patients [26]. We developed and delivered a virtually facilitated AGF session based on the CAFF approach for Internal Medicine and Hospitalist (Family Medicine trained) physicians across four tertiary care hospitals in Calgary, Canada between January to June 2021. The AGF sessions were part of a multifaceted intervention bundle to safely reduce repetitive use of routine laboratory testing in hospitals [26]. This bundle included an online educational module, educational decision-aids and posters, and an individual physician report with peer comparators that was electronically delivered to each physician every 2 months during the intervention period [26]. The educational materials and individualized reports were shared through secure email links prior to the session [26].

Virtual AGF sessions were developed and delivered by the physician leads (DW, AA) and project managers (AM, LF). DW and AA had prior experience delivering in-person AGF sessions using the CAFF approach. The CAFF describes three prerequisites before participants can move through the sequence of behaviours towards planning for change [15]. The first prerequisite is relationship building which refers to the process of establishing trust and respect between facilitators and participants, with an emphasis on education rather than assessment [11, 15]. The second prerequisite is the question choice, which must be important for patient care, actionable, and under direct control of the participant [11, 15]. The third prerequisite is data representation which considers that the data is meaningful and easy to interpret for participants [11, 15]. While completing prerequisite activities, results from a prior successful pilot study were reviewed, in which a decrease in lab ordering had been seen [27].

The format, timing, use of tools, and role of facilitators was planned to reproduce the elements of CAFF during virtual AGF sessions. We used Zoom [28] as our virtual videoconferencing platform with the breakout room functionality for brainstorming and discussion in small groups of up to 6 participants paired with a trained facilitator. Participants were encouraged to turn on their cameras and microphones in breakout rooms. Participants were able to access a live video of the sessions on a desktop, laptop or mobile device, and were able to participate online with audio, video, chat, or by telephone. We facilitated participant input using Mentimeter (an online polling tool) for polling and real-time visualization of participant responses in word cloud format [29].

We used a retrospective mixed methods approach to conduct a process evaluation to assess if the virtual AGF intervention was developed and delivered with fidelity to the CAFF approach. We reviewed study documents and session artifacts including meeting minutes, field notes, Mentimeter [29] results, session summaries, and post-session team debrief notes to assess fidelity to the CAFF approach in the development and delivery of the virtual AGF sessions. Recordings of the sessions, Zoom [28] chats, and Mentimeter [29] polling results were transcribed, time-stamped, attributed to de-identified peer facilitator, non-physician facilitator from the Physician Learning Program [25], or participant and organized in an excel spreadsheet. Team members (AA, DW, LF, OO) familiarized themselves with the de-identified data through iterative reading and discussion. We created a coding framework based on the conceptual model of physician behaviours described during in-person CAFF-based AGF sessions [15]. The codebook (Appendix 2) included the three CAFF prerequisite activities of relationship building, question choice and data representation, along with the anticipated sequence of participant behaviours with descriptions and examples to ensure analytical coding consistency. We used NVivo 12 Pro software [30] to code transcript sentences, Zoom [28] chats, and Mentimeter [29] results and data, could be coded to multiple nodes. After individual coding was complete, inter-rater reliability was calculated and sentences with low inter-rater reliability were reviewed, discussed, and re-coded if appropriate. Final Cohen’s kappa values were between 0.95–1.00 indicating excellent agreement between coders [31]. Quantitative analysis of results was conducted using simple descriptive statistics.

## Results

Twelve virtual AGF sessions were conducted with 83 physicians. Eight initial sessions were conducted across the four sites (one session per site per specialty i.e. Internal Medicine or Hospitalist), with four follow-up sessions conducted at each site. This process evaluation focuses on the eight initial sessions that were based on the CAFF approach. Initial sessions 3, 5 and 7 had to be excluded from some quantitative analyses describing percentages due to technical limitations that impacted data quality from recordings. However, they were included in the qualitative analysis based on availability of other study documents including field notes. Table 1 highlights the differences in the conduct of in-person AGF sessions versus the virtual session. Table 2 describes the operationalization of the three CAFF prerequisites in the design and delivery of AGF sessions. Once the prerequisite activities were completed, participants were given individual brainstorming time to think of change ideas and then assigned to virtual breakout rooms to discuss these ideas, in a maximum ratio of six participants to one facilitator. After the breakout sessions, facilitators from each group summarized the change ideas generated with all the session participants. The virtual AGF sessions concluded with a discussion around prioritization of change ideas and physicians were encouraged to complete an online survey with a personal commitment to change (which asked them to commit to a specific change action, date of change, and expected benefits), and a session evaluation (Appendix 3).

**Table 1:** Comparison of in-person and virtual Audit and Group Feedback sessions (AGFs)

	In-person AGFs	Virtual AGFs
Format	• On-site meeting room with up to 20 participants in-person • Participants usually from a single geographic site • Lunch/snacks provided • Informal conversations prior to start of session to build relationships and engagement	• Zoom meeting with up to 25 participants • Participants from multiple geographic sites • Participants mostly individual, but sometimes in groups of 2–3 on a shared device and occasionally in larger groups using meeting room video-conferencing equipment • Polling used at beginning of the session to increase engagement
Facilitators	• PLP^a^ medical director and a physician lead co-facilitate sessions • PLP^a^ project manager to help with room set-up and flip charts	• PLP^a^ medical director and physician lead co-facilitate sessions • PLP^a^ project manager supports overall virtual session • Each virtual breakout room is facilitated by a trained PLP^a^ facilitator
Agenda	• 1 h • Less structured agenda • At least 50% of session in group discussion • Agenda more reflexive to the needs of participants • Usually followed by informal discussion among about half of participants at the completion of the session	• 1 h • Very structured agenda • At least 50% of session was spent in virtual breakout rooms/discussion • 'Ghost’ participants were commonly identified when participants were assigned to breakout rooms and encouraged to turn on cameras and microphones (assumed to be people logged but not actually present) • Interaction during plenary presentation encouraged through online polling, with minimal verbal contributions from participants during plenary • Virtual breakout rooms to facilitate more informal discussion with a maximum of 6 participants per breakout room, each hosted by a trained facilitator
Reports	• Hard copy confidential practice reports provided to participants • Usually reviewed by participants prior to starting the formal session	• Link to confidential practice report provided to participants • Unclear how many participants accessed these prior or during the AGF sessions
Facilitation tools	• PowerPoint presentations prepared but rarely presented as participants had hard copy reports to refer to • Flip charts or physical whiteboards to document key discussion	• Videoconferencing hardware and software (Zoom) • Presentation software (Powerpoint) • Polling software (Mentimeter)
Closing	• Participants are handed a paper copy of the Commitment to Change and Evaluation Form that they complete before leaving • Completion rates for both are usually close to 100% • Some participants often continue discussion after the formal closing	• Participants had to scan a link with their phone or type in a web address to an online survey for Commitment to Change (CTC) and Evaluation Form. Participants were given time before the end of the session to complete these • Completion rates were 46% (38/83) for personal CTC and 66% (55/83) for session evaluation • Rare that participants stayed online after the completion of the formal session to provide feedback or engage in further discussion

^a^ *PLP* Physician Learning Program

**Table 2:** Operationalization of the three CAFF prerequisites in the design and delivery of the virtual AGF session

CAFF Prerequisites	Design	Delivery
1. Relationship building	Peer facilitators **:** Both physician peer facilitators worked at participating hospitals and understood the local context, were familiar with the data and had existing relationships with many of the participating physicians	All our virtual AGF sessions, including breakout room discussions were facilitated by at least one of our physician peer facilitators (DW, AA)
	Site champions: One internal medicine site champion and one hospitalist site champion at each hospital were identified The site champions helped co-create the AGF session and led recruitment at their site A “Train the Trainer” session was conducted prior to the AGF session, with site champions to increase their familiarity and comfort with the CAFF approach and to build facilitation skills	Site champions agreed to share their personal data and reflect on practice change opportunities during virtual AGF sessions Site champions facilitated Breakout rooms discussions when needed. Breakout rooms were facilitated preferentially by peer facilitators, then by site champions, then by non-physician facilitators from the Calgary Physician Learning Program
2. Question choice	Pre-session handouts: Prior to the sessions, participants were emailed information on low-value care, such as the Choosing Wisely Canada’s recommendations on low-value laboratory testing, along with their individualized reports Effort was made to ensure that lab ordering data was attributable to individual physicians and that metrics represented ordering behaviours under the direct control of physicians	Participants were asked to respond to the question: “Why is it important to improve lab ordering?” A case study was presented to encourage reflection on lab ordering behaviour in the context of a clinical scenario Recommendations and evidence for laboratory test ordering were presented Results of successful pilot study [27] led by AA, which resulted in decrease lab ordering, was reviewed
3. Data representation	Evidence-based metrics: Data metrics were selected based on relevance, interpretability, and data availability. The pilot study [27] informed the selection of three primary metrics: 1. number of routine tests ordered per patient-day, 2. cost of test ordering and 3. number of test-free patient days Physician leads identified and discussed practice data validity and limitations while designing the session Reports showed individual results compared to their anonymized peers. No specific targets for lab utilization were provided An online dashboard with individualized reports was created using a human-centered design approach, and participants received a pdf version of their individual report by secure email approximately 10 days prior to AGF sessions	During the session, the peer facilitators shared their own reports and discussed implications on their practice, including questions and concerns with data validity and limitations

**Fig. 1:**
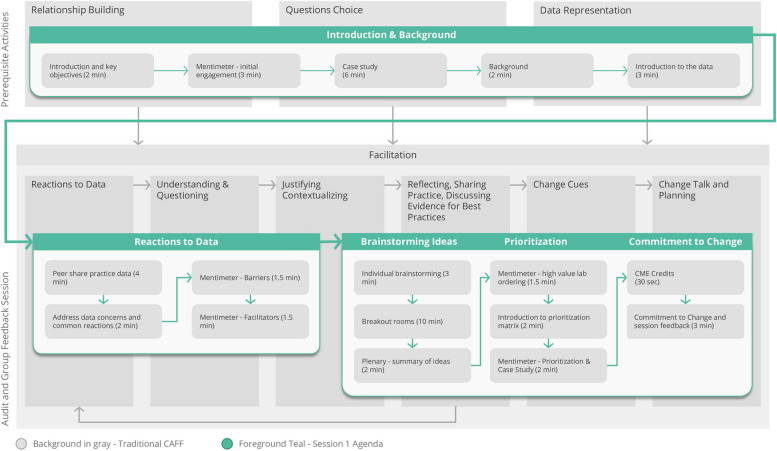
Mapping of the session design to the different elements of CAFF along with the time allotted for each for a one-hour facilitated session. CTC: Commitment to Change

**Fig. 2:**
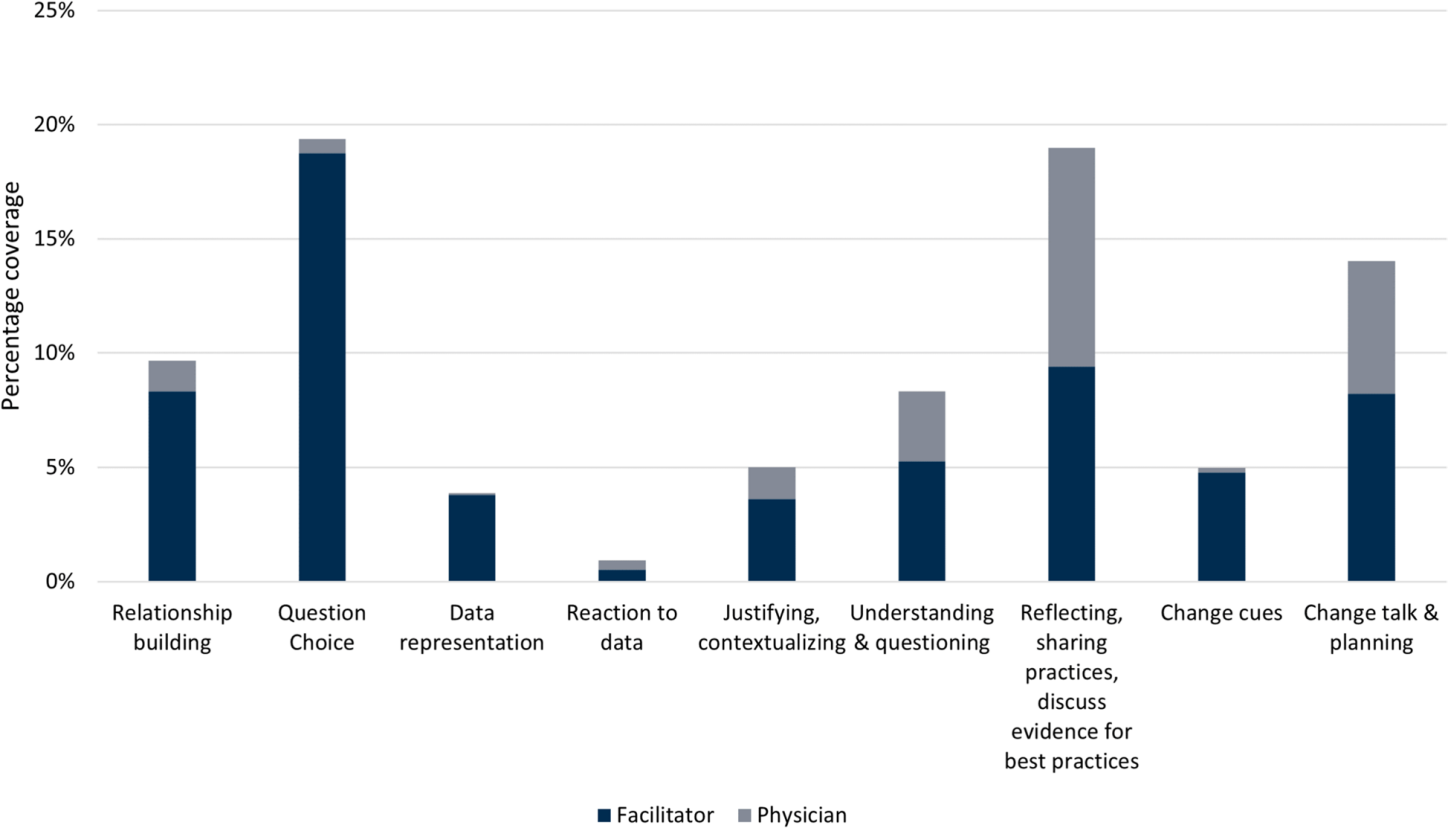
Facilitator and physician participant contributions to CAFF elements for virtually facilitated AGFs

**Fig. 3:**
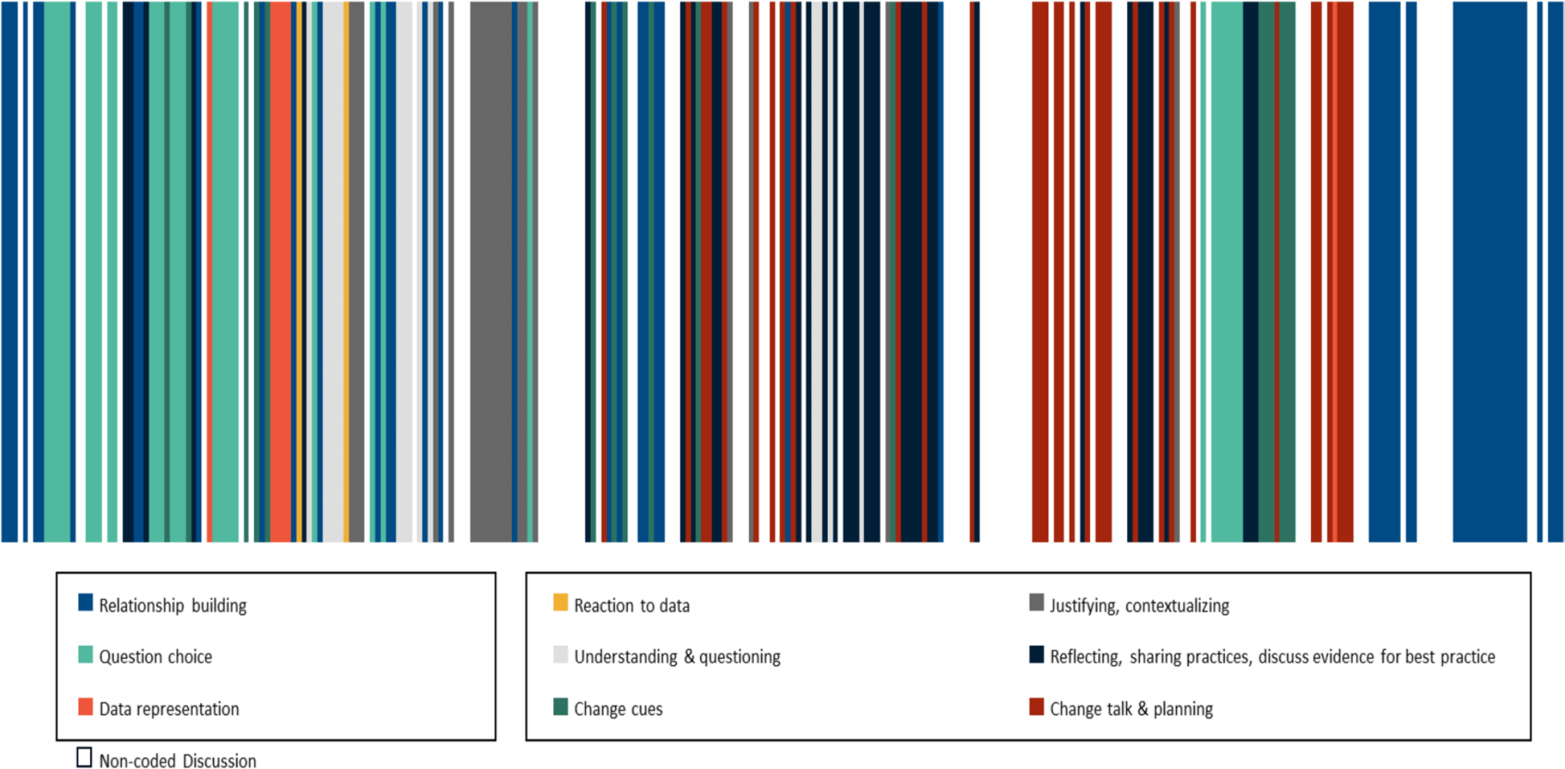
Frequency of CAFF elements over time for Session 1 demonstrating the non-linear and iterative version of discussion

Figure 1 displays how our session design, beginning from ‘Introduction’ and then moving to ‘Reactions to Data’, ‘Brainstorming Ideas’, ‘Prioritization of ideas’ and finally ‘Commitment to Change’ were mapped to the different elements of CAFF within a one-hour session. Figure 2 demonstrates that the prerequisite activities of relationship building, question choice and data representation were discussed in every session, as were participant behaviours for progression towards change talk and planning. Both peer facilitators and participants contributed to the CAFF elements. All participants contributed to discussion during the breakout rooms and representative quotes from participants about change talk and planning are included in Table 3. The discussion during one representative virtual AGF session was analyzed in detail in Fig. 3. Although the AGF agenda was based on a linear progression through the prerequisite activities and participant behaviours, our analysis demonstrates that these elements occurred in a non-linear and iterative manner during AGF delivery. The participating physicians not only interacted with their data but were emotionally engaged during the virtual AGF session as exemplified with the following quote:

*“I think understanding the harms to the patient of doing labs. So, when we have those poor longer-term patients who have anemia from our repeated bleeding or the fact that you just need to think about it, they're a tough poke, it's painful to do this, does this really need to happen today? I think it's a number on a screen and we forget the person getting the actual needle in their arm each day.”*

**Table 3:** Representative quotations from participants that display the behavior of “change talk and planning”

Session Number (Participant ID)	Quotation
Session 1 (Participant 4)	“… asking yourself what you're expecting to see and how it'll change management. Sort of the same thing that we're asking our residents to do sometimes we're guilty of the same thing, just by not reflecting enough before ordering, so just being vigilant I guess.”
Session 2 (Participant 3)	“…if you're ordering a test several days in a row and it hasn't changed, maybe there can be some kind of smart alert that says, you know this hasn't changed in three days do you really need it?
Session 3 (Participant 1)	“People that are in the hospital for months, do they really need to be on low molecular heparin? You know, start thinking about as I guess it's a sidebar to lab utilization is med utilization. Because if somebody is on an unnecessary medication that ends up generating unnecessary tests. It's a double whammy.”
Session 4 (Participant 4)	“I am almost thinking giving a justification every time you order a lab, what are you looking for, and that can either work well or badly, certainly I notice that a lot of labs are noticed for no obvious reason, if there was just a valid reason for why a test was being ordered, that's my idea”
Session 5^a^ (Facilitator)	“…if you had more assurance that if you order labs later in the day and they would happen on a timely basis, you wouldn't be as concerned about doing it first thing in the morning
Session 6 (Participant 2)	“And perhaps that needs to be more explicitly written into our daily notes or progress notes, or the delivery during rounds is to include…We don't necessarily think of labs as an intervention but perhaps we should.”
Session 7^a^ (Facilitator)	“Treat the patient, not the numbers. Make it clear why the test is being ordered in consultation letters. Lab checklist or stickers (“do they need labs tomorrow?”) – prompt to think about this at the end of the note.”
Session 8 (Participant 9)	“More mindful lab ordering, decreased risk of anemia”

^a^Session 5 and 7 are ideas generated by participants in breakout rooms, summarized by facilitators

66% (55/83) of the participants filled out the post AGF session survey. 98% of responding physicians (54/55) reported that they would recommend the program to a colleague. 94% (46/49) of those that responded to this question reported that participating in the program helped them improve their practice. 38 of the 83 participants (46%) completed the commitment to change form.

## Discussion

We demonstrate that virtual AGF sessions, developed and implemented with fidelity to the CAFF approach, successfully engaged groups of physicians in change planning. In a separate evaluation, these AGF sessions, as a component of a de-implementation strategy and multi-faceted intervention bundle resulted in a 14% reduction in ordering of common laboratory tests [26]. A prior study using the CAFF approach with in-person AGF sessions also showed a similar decrease of around 15% in low value care for bronchiolitis patients in the Emergency Department [16]. Target behaviours for inpatient laboratory testing are poorly defined due to the heterogeneity of the tests and lack of a ‘gold standard’ solution [6]. It is an important finding that virtual AGF, delivered with fidelity to the CAFF model, and without reference to specific laboratory test ordering targets, successfully moved participating physicians through a cycle of behaviours leading to change talk and planning and was associated with a reduction in laboratory test ordering [15, 32].

AGF peer facilitators, both of whom worked as physicians on general medicine and hospitalist wards, helped advance the agenda towards planning for change. This is consistent with the emphasis on facilitation by a respected peer with an understanding of the contextual nuances of the topic that is described by the CAFF for in-person AGF sessions [33, 34]. Our peer facilitators shared their own reports to discuss its implications on their practice as a motivator to facilitate sustainable change in established behaviour of colleagues [11, 33]. In AGF sessions, the peer facilitators addressed barriers to feedback use in a social environment, which is important to optimize the effect of feedback on practice change [11]. We kept our data simple with three primary metric measurements and used clear graphs to minimize extraneous cognitive load for recipients, another recommendation to optimize effectiveness [11].

Our study advances our knowledge and application of CAFF for AGF sessions. Our findings support the inclusion of CAFF prerequisite activities in AGF agendas regardless of effort spent on these activities prior to sessions. We demonstrate that facilitator and participant discussion during AGF sessions was non-linear despite the agenda being based on a linear progression through CAFF elements. There were frequent jumps forward (e.g., discussing change planning very early in AGF sessions) and backwards (e.g., reviewing question choice near the end of AGF sessions). We believe this non-linearity is a facilitation technique to support participants to progress towards change talk and planning while still providing opportunities to review previous CAFF elements when necessary. We perceive benefits of delivering virtual AGF sessions compared to in-person AGF sessions including easier scheduling, higher participation rates, greater geographic access, and reduced travel time. Furthermore, our study provides context-specific insight into de-implementation of the practice of repetitive laboratory testing.

### Limitations

Recording quality of sessions 3, 5, and 7 was inadequate for complete and accurate transcription, thus these sessions could not be included in our quantitative analyses describing percentages by character count. However, we reviewed and coded available notes and portions of transcripts to ensure that our results still apply to those sessions. Physicians who chose to attend AGF sessions may have been more willing to engage in discussion and move through our agenda than their non-attending colleagues. Further study to identify the most effective ways to engage ‘unwilling’ participants may advance our understanding of change management. In our prior experience we have been able to conduct in-person AGF sessions with one lead facilitator and one site lead physician for groups of 20 physicians at a time. During virtual AGF sessions we limited breakout rooms to a maximum of six physicians to encourage interaction, each with one trained facilitator from our team. This increased the number of skilled facilitators needed compared to in-person AGF session which is an important resource consideration in adoption of virtual over in-person AGF sessions. Further study to evaluate the impact of virtual AGF sessions on topics other than laboratory test utilization would help clarify the extensibility and limitations of this approach for other topics. Virtual AGF may not be as effective for complex or controversial topics, particularly if limited social interaction impedes discussion about potential unintended consequences of change ideas. We were not able to prove which components of our AGF intervention were most effective in creating a social learning environment leading to practice change. For example, the non-linearity that we demonstrated in program delivery suggests that skilled facilitators play an active role in adapting the agenda to the perceived needs of their audience during AGF delivery. Further study to identify how much AGF sessions can be standardized without compromising impact, and which AGF components are the most effective in promoting practice change, may help to reduce dependence on skilled peer facilitators and increase opportunities to spread and scale AGF interventions.

## Conclusion

Virtual AGF sessions based on the CAFF were successful in engaging groups of physicians in change planning and as a core component of a multifaceted intervention bundle, resulting in a change in physician laboratory ordering behaviour.

## Supplementary Information

Supplementary Material 1.

Supplementary Material 2.

Supplementary Material 3.

## Data Availability

The datasets used and analyzed during the current study are available from the corresponding author on reasonable request.
