# Supplementary material for: Extending the Calgary Audit and Feedback Framework into the virtual environment: a process evaluation and empiric evidence
Source: Implement Sci Commun. 2024 Dec 18;5:140. doi: 10.1186/s43058-024-00679-5 (PMC11657922; doi:10.1186/s43058-024-00679-5)
Supplement: Supplementary file 2 — Supplementary Material 2. [file 43058_2024_679_MOESM2_ESM.docx]

Appendix 2: Codebook

| **Parent Node** | **Child Node(S)** | **Description / Notes** |
| --- | --- | --- |
| CAFF Pre-requisite activities | *Relationship Building* | Establishing **trust** and respect between A&F providers, facilitators and participants.  Education rather than assessment focus.  Empowering a group member to co-facilitate the AGFs, clarifying use of and the confidential nature of the AF reports, and the data limitations. |
|  | *Question Choice* | Is the topic important to participants and patient care?  Is it actionable?  Is it under direct control of physician?  Are data sources and limitations clear?  Availability of best practice evidence or gold-standards?  Is there a problem?  What is the problem? |
|  | *Data representation* | Is the A&F report easy to understand and interpret?  How data is defined, used, formatted. |
| Conceptual Model of Physician Behaviours | *Reactions to data* | Ranging from satisfaction to skepticism.  Anything emotional.  Reactions to participant’s own data and own report specific around their lab ordering. |
|  | *Justifying, contextualizing* | Trying to identify potential explanations relating to personal, patient, system, or other factors.  Specific to participant’s ordering practices, why they do things the way they do.  More on the defensive, participant’s defending their current practice rather than thinking about new options. |
|  | *Understanding & questioning* | Understanding data, including limitations of data.  Anything about questioning, understanding, or improving. |
|  | *Reflecting, sharing practices, discuss evidence for best practice* | Reflecting on data by sharing findings of their own reports and their personal experiences and practices.  Participants talking about what they do, pure reflection.  Reflecting and sharing applies to both participant and facilitator. |
|  | *Change cues* | Turning points in the group discussion, initiated by a brief comment highlighting the importance of a performance gap revealed by the data reports.  Raised spontaneously by a participant or facilitator.  “aha” moment  Identifies something we need to fix, change, do better, or do differently. |
|  | *Change talk & planning* | Discussion of how a care gap could be addressed, what would need to be done, and by whom.  Specific idea, plan, practice change idea.  Thinking about and prioritizing the change ideas. |
