# Supplementary material for: Extending the Calgary Audit and Feedback Framework into the virtual environment: a process evaluation and empiric evidence
Source: Implement Sci Commun. 2024 Dec 18;5:140. doi: 10.1186/s43058-024-00679-5 (PMC11657922; doi:10.1186/s43058-024-00679-5)
Supplement: Supplementary file 3 — Supplementary Material 3. [file 43058_2024_679_MOESM3_ESM.docx]

Appendix 3: Evaluation Survey Questions

1. What is one thing we should stop doing or that you disliked about our Audit and Feedback Session? Use the textbox below to answer.
2. What is one thing we should keep doing or that you found really useful during our session? Was there a particular highlight of the session? Use the textbox below to answer.
3. Would you recommend this session to a colleague? Yes/No

Use the 5-point Likert scale below to answer the following four statements (4-7):

1. The report helped me to improve my practice (e.g. provide higher quality of care, increase efficiency)
2. The workshop helped me to improve my practice.
3. Overall, participating in this program helped me to improve my practice.
4. How would you rate this session?
5. Did you complete a commitment to change form? Yes/No
6. Do you have any other comments/suggestions? Use the textbox below to answer.
